# Multiple Sclerosis: A Global Concern with Multiple Challenges in an Era of Advanced Therapeutic Complex Molecules and Biological Medicines

**DOI:** 10.3390/biomedicines6040112

**Published:** 2018-11-30

**Authors:** Victor M. Rivera

**Affiliations:** Department of Neurology, Baylor College of Medicine, Houston, TX 77030, USA; vrivera@bcm.edu; Tel.: +1-832-407-0668

**Keywords:** multiple sclerosis, genetics, disease modifying therapies, generic medicines

## Abstract

Multiple sclerosis (MS) has become a common neurological disorder involving populations previously considered to be infrequently affected. Genetic dissemination from high- to low-risk groups is a determining influence interacting with environmental and epigenetic factors, mostly unidentified. Disease modifying therapies (DMT) are effective in treating relapsing MS in variable degrees; one agent is approved for primary progressive disease, and several are in development. In the era of high-efficacy medications, complex molecules, and monoclonal antibodies (MAB), including anti-VLA4 (natalizumab), anti-CD52 (alemtuzumab), and anti-CD20 (ocrelizumab), obtaining NEDA (no evidence of disease activity) becomes an elusive accomplishment in areas of the world where access to MS therapies and care are generally limited. Countries’ income and access to public MS care appear to be a shared socioeconomic challenge. This disparity is also notable in the utilization of diagnostic tools to adhere to the proposed elements of the McDonald Criteria. The impact of follow-on medications (“generics”); injectable non-biological complex drugs (NBCD), oral sphingosine-1-phosphate receptor modulators, and biosimilars (interferon 1-a and 1-b), utilized in many areas of the world, is disconcerting considering these products generally lack data documenting their efficacy and safety. Potential strategies addressing these concerns are discussed from an international point of view.

## 1. Introduction

Multiple sclerosis (MS) is an inflammatory and demyelinating disease that manifests pathologically and clinically after the disruption of the dynamic equilibrium of brain plasticity enables the development of a chronic process affecting the central nervous system (CNS). Common association with comorbidities impacts the course of disease and quality of life of the individual. MS derives from a complex multifactorial etiological process where genetic and environmental agents decisively interact. Neuroinflammation associated to MS results in a constellation of clinical manifestations as well as mood disorders, depression, and anxiety in a large proportion of patients [1]. Persistent inflammation is also one of the causes of chronicity of disease and phenotype definition [2]. The disease may become neurodegenerative, progressive, and incapacitating in almost of half of the untreated population [3]. This outcome has been improved by early and effective use of disease modifying therapies (DMT [4]. The disease commonly affects white Caucasians, particularly people of Northern European ancestry and their descendants living in recognized high-risk areas of the world: Scandinavia and the British Islands, Canada, the U.S., Australia, and New Zealand. Nevertheless, MS is increasingly identified among populations who were considered uncommonly affected by the disease. This phenomenon is generally attributed to genetic dissemination from high- to low-risk groups owing to historical and political events favoring racial intermixing. This situation has apparently contributed to the increasing frequency of the disease among Latin American Mestizos and African Americans [5]. Similar observations apply to Māori people in New Zealand, whose present genetic make-up is described as of both European and aboriginal descent [6]. Higher MS frequency rates have been reported recently in Middle Eastern and North African countries [7,8], while in other areas of the world (Asia, South America), serial epidemiologic studies reveal a true augmentation in regional rates occurring over short periods of time [9,10]. Other factors contributing to the globalization of MS are exposure to changing environmental factors, improved medical education on the disease, increasing availability of neurologists in most areas the world, as well as magnetic resonance imaging (MRI) machines, and widespread public awareness, including locally developed patient support groups and coordinated international advocacy groups like the MS International Federation (MSIF, London).

The increasing presence of MS has resulted in serious challenges to providing adequate care and accessibility to therapies. The socioeconomic challenges posed by MS as a universal disease are emphasized in countries with economies in development, but it is also an important consideration in industrialized countries that theoretically have more advanced health systems.

From initial diagnosis to long-term management, MS is a very onerous and complicated medical condition. The disease exerts a substantial economic impact on health systems, particularly where therapeutic availability is compromised by technological limitations to fulfilling all necessary elements for diagnosis proposed by modern criteria. The impact of follow-on “generic” and biosimilar medications in some areas of the world deserves discussion in view of the lack of data substantiating their efficacy and safety profiles. These preoccupations are enhanced in many areas of the world where limited capabilities exist affecting their local licensing agencies in their ability to provide an objective, analytical, and educated approval process for complex therapeutic molecules.

This commentary addresses the concerns derived from the expanding global presence of MS, the unexpected consequences of the socioeconomic burden to MS communities, and the impact exerted in the different aspects of the disease, from adequate application of the elements of the current diagnostic criteria to access to care. Potential alleviating strategies are discussed.

## 2. The Global Emergence of MS

Following Jean Martin Charcot’s papers on his lessons on “La Sclèrose en Plaque Disseminées” in 1868 [11], scholars in France and Europe utilized the modified denomination “Insular sclerosis of the Brain and Spinal Cord”. The term “The Multiple Scleroses (as utilized in the paper) was first employed by the Philadelphia botanist Horatio Curtis Wood in 1878 [12] and adopted internationally since then as multiple sclerosis. For decades, European and American clinicians considered it as a “new” but rare neurological disease studied merely in the U.S. and Western Europe. The perception that MS was minimally or non-existent in places with non-Caucasian populations was reinforced by the 1970 observation from Alter and Olivares [13] on the prevalence in Mexico as “one of the lowest in the world” (1.6/100,000). During the last part of the 20th century and the first decades of the current epoch, epidemiologic studies have shown a notable increase in prevalence in Latin American countries [14], including Mexico [15], and the Middle East [16], while frequencies remain elevated in North America and some European countries. On the American continent, the increasing presence of the disease is now evident in populations that were hypothetically “resistant” to the disease. For five centuries, historical, sociopolitical, and migratory events favored the introduction of the European genetic risk into Native Americans (or Amerindians) and into Central and West African groups brought to the continent between the 16th and 19th centuries, resulting in the modern emergence of MS among the Latin American populations [17]. Mestizo groups constitute the most representative ethnic group in Latin America and form the largest minority in the U.S. (“Hispanics”). Studies consistently show these groups carry the inherited MS genetic European signature: HLA-DRB1*1501 [18,19]. On the other hand, the disease is rare, or practically non-existent, among non-mixed Amerindians [20]. The most plausible explanation for this phenomenon lies in the fact that Native Americans (across the continent) possess a predominantly Asian genetic makeup probably owed to the early peopling of the Americas. Low prevalence continues to be reported among Chinese communities (5.2/100,000) [21], in Japan (3.9/100,000), and in Korea (3.5/100,000) [22,23]. Contrarily, Western Siberian populations have increased their prevalence in the last thirty years from 24 to 54/100,000 [24]. It is noted the Western Siberian MS patients are practically of European origin (white Caucasians). The disease however remains unreported among Yakuts and smaller Asiatic tribes [25]. At present, the MS prevalence in the Russian Federation is at a medium risk level (30–70/100,000) [26].

Despite epidemiologic methodological inconsistencies in acquiring data in the Middle East and nearby areas, current information shows frequencies fluctuating from low to high prevalence in this region [27]. Substantial MS prevalence has been noted in some countries, i.e., the United Arab Emirates 64.4/100,000 [28] and Iran 101.13/100,000 [29]. Observations in Kuwait show Palestinian emigres have a higher prevalence (23.8/100,000) in comparison to local Kuwaitis (9.5/100,000) [30]. Qatar reports a high MS concentration (64.57/100,000), also contributed in part by a large immigrant working force [31].

The highest prevalence rates are reported from the Scottish Northern Isles: the Orkney (402/100,000) and Shetland Islands (295/100,000) [32]. Prevalence in mainland Scotland is very high as well: 229/100,000 [33]. Canada claims the highest national prevalence at 290/100,000 [34]. The prevalence in U.S. has been reported with varying rates: 110 to 192.1/100,000, from the Eastern and Western census, respectively [35]. The majority of global MS epidemiologic studies address prevalence whilst international incidence studies are scarce. Nevertheless, the MS world map exhibits frequent and dynamic changes as more epidemiologic data accumulates from the different regions of the globe.

## 3. Ubiquitous Application of MS Diagnostic Criteria

The criteria for diagnosing MS have evolved along with advances in knowledge of the disease. The process of diagnosing MS following an initial clinical event, or clinically isolated syndrome (CIS) suggestive of an inflammatory/demyelinating lesion, or lesions, in the CNS, has become more sophisticated, while concomitantly, the international panel authorizing the criteria strive for simplicity and general accessibility of the proposed guidelines. The 2017 McDonald Criteria [36] was designed to serve as a more accessible tool for practitioners and researchers for reaching a faster and more definite MS diagnosis. The criteria aim to increase sensitivity without affecting specificity, reducing the possibility of misdiagnosis, and adding novel aspects in its structure, like the inclusion of symptomatic and asymptomatic lesions, as well as cortical signals detected by MRI, to comply with the concepts of lesions disseminated in space (DIS). Another original addition introduced by the 2017 McDonald international panel is utilizing the presence of unique cerebrospinal fluid (CSF) oligoclonal bands substituting for dissemination in time (DIT) in cases lacking MRI asymptomatic post-gadolinium T1 enhancing images. Assessment of optic pathology, although important, is not included within the current stipulations of the 2017 McDonald Criteria.

Factors affecting realistic applicability of the criteria in all areas of the world are related to limited access to diagnostic technology or to economic constraints. The MSIF reports an increasing trend in the number of MRI machines in emerging countries, almost doubling in a five-year period. Still, 87% of low-income countries [37] do not use the McDonald Criteria reporting criteria, instead utilizing the outdated Poser criteria (1983) [38] which does not require MR imaging for the clinical diagnosis of MS. Another aspect determining effective universal applicability of the criteria is the fact that the most sensitive and recommended methodology for CSF oligoclonal bands analysis, isoelectric focusing immunoblotting [36], is not readily available through local clinical laboratories in countries with developing economies. This technique requires special equipment and expertise to perform the analysis. The older techniques, i.e., agarose gel electrophoresis, are less sensitive and carry substantial risk of providing false-positive results. Many neurological communities in regions facing this dilemma have opted to omit CSF analysis in the diagnostic workup of suspected MS.

The McDonald Criteria panel recognizes that the proposed elements for diagnosis have been acquired from large populations of Western European genetic origins presenting with typical CIS (the initial MS clinical event). The panel emphasizes the need to validate the criteria, either prospectively or retrospectively, in diverse populations, namely in patients from Asia, Latin America, the Middle East, Africa, and other relatively less studied geographical locations. Recent discussions at the Foro Centroamericano y del Caribe para Esclerosis Múltiple (FOCEM) [39] addressed the difficulties in fulfilling the diagnostic criteria in some areas of the world. FOCEM is constituted of neurologists from the six Central American nations, Venezuela, and 26 Caribbean island countries. Most neurological services in these countries have access to MRI, and mostly to 1.5 Tesla equipment; however, practically none of these diagnosticians possess reliable CSF analysis technology to locally perform complimentary analyses. The risk of underdiagnosing in areas of the world where these limitations exist is a realistic concern [40].

It is expected that future availability of economic serological biomarkers will considerably alleviate the diagnostic restrictions existing for MS in some areas of the world.

## 4. Global MS Care Disparities

MS therapies have flourished in the last three decades whilst becoming more complicated and onerous. The advent of what are recognized as high-efficacy medications applies mostly to DMT for relapsing MS (RMS), with thus far only one MAB approved for primary progressive MS (PPMS). These medications have a greater pharmacological effect than the original first-line, platform, injectable therapies (interferons and glatiramer acetate). International licensing agencies, satisfying an unmet therapeutic need, have approved ocrelizumab, a CD20 cytolytic MAB targeting B lymphocytes, for treatment of PPMS. Several clinical trials are being carried out addressing progressive forms including secondary progressive MS (SPMS). However, making these pharmacological agents accessible to all MS populations is a formidable challenge and realistic socioeconomic preoccupation. Except for the private health enterprise sector of the U.S., most countries of the world rely almost entirely on their official health systems to provide access to MS therapies. For most of the 101 countries that provided data to the MSIF, therapies were partially or fully funded by the government. In the countries affiliated to the MSIF, health services funded by taxation through social security or mandated health insurance covered 76% of the cost of DMT. Global availability of MS medications is notably dissimilar between high-, upper middle-, lower middle- and low-income countries, as described in the Atlas of MS (World Health Organization/MSIF) [41]. The higher the national income, the more availability of medications—not just platform injectable medications, but oral agents and intravenous MAB, as well. Most countries with the lowest national incomes may have access to only one or two first-line DMT. For instance, in Cuba, the only DMT available is the brand Interferon beta 1-a, 44 mcg (Rebif^®^) [42]; in the Republic of Salvador, the national social security system offers only two medications, both innovators, including a low-dose (Avonex^®^, 30 mcg) and a high-dose interferon beta 1-a (Rebif^®^, 44 mcg) [43]. Availability of DMT to MS patients in the world is reviewed in detail at the Atlas of MS 2013, with data provided by the World Bank and the World Health Organization [44]. Availability of medications for all people with MS is not a reality for at least 90 countries of the world. The MSIF document indicates that affordability was ranked as the most common cause of lack of access to therapy in 46% of countries, which rises to 86% in 21 low- and lower-middle-income countries.

The socioeconomic impact of MS in developing countries is a considerable public health concern. These same areas usually display a low prevalence of MS; hence, the disease is not generally appreciated by their health systems. In economically emergent countries in Latin America, for instance, only 9.5% to 42.3% of the MS population have access to a DMT [45]. On the other hand, almost 90% of the economic burden exerted by MS on the country of Colombia is spent just to cover the cost of DMT [46], this cost being dependent on the grade of disability: For a patient with Expanded Disability Score Status (EDSS) 3.0-5.5, the annual cost of MS medications is 25,713 USD, while for a patient with EDSS ≥7.0 in Argentina, it is estimated at 50,712 USD. In general, the cost of DMT is less expensive in European countries and elsewhere, in comparison to the United States where, for example, an interferon for Relapsing Remitting MS (RRMS) may cost as much as 5150 USD/month, and oral fingolimod 5372 USD/month. Other tangible and intangible costs, including medical expenses, rehabilitation procedures, other multidisciplinary care required by MS, loss of work productivity, and the emotional and physical impact on the care givers, add exponentially to the price of MS. The cost increases as disability advances [47]. In many countries where access to DMT is limited, escalation to drugs with higher efficacy is not feasible; hence, the ideal goal of obtaining the therapeutic goal of ‘no evidence of disease activity’ (NEDA) is consequently and fundamentally challenged. Potential strategies to address these concerns would involve increasing public awareness and knowledge, which eventually should also impact health officials’ education and attitudes. Transparency in the process of MS medication acquisition by national health systems would be more efficient and cost-containing by involving neurologists with MS knowledge and independently appointed public commissioners with input from patients support groups in this complex undertaking.

## 5. Impact of Follow-On Therapeutic Molecules and Biosimilar Medications

The appearance of “generic” medications for MS in international public and private health markets, and prescription formularies of social security programs around the world, has been increasing in development. International regulatory agencies, including the U.S. Food and Drug Administration (FDA) and the European Medicines Agency (EMA), have approved follow-on CBND replacing the innovator Copaxone^®^. This decision was based on bioequivalence shown by molecular and pharmacological similarity, without requiring clinical studies. Neither the FDA nor EMA have determined approval pathways for follow-on biomedicines such interferons and MAB. In both cases, appropriate phase III clinical data, and even “head-to-head” trials performed on the proposed follow-on medication set against the innovator drug as a substitute, should be required by international licensing agencies. The lack of essential clinical and pharmacological data from biosimilar medications is reflected in the fact in that most international MS associations do not include or consider them as yet in their therapeutic guidelines: These include the American Academy of Neurology, the European Academy of Neurology, the Spanish Society of Neurology, the Catalonia Society of Neurology, Consensus from Peru, Central America and Caribbean countries, among others. Some of the follow-on products manufactured in North Korea, India, Iran, Mexico, Argentina, and Uruguay (outside the sphere of the FDA and EMA), lack data on efficacy and safety of their own, in fact utilizing results obtained from the phase III pivotal trials performed by the innovator (original) products. Follow-on medications have been approved by many international licensing agencies outside the U.S. and the European Union, and are basically unchallenged due to lack of local appropriate technology and education on the subject of the responsible health departments, including the ability to evaluate the biological and immunologic behaviors of the proposed product. Substantial molecular differences have been reported [48] between follow-on and innovator interferon drugs. Analytical studies performed on the interferon 1-a innovators Avonex^®^ (30 mcg) and Rebif^®^ (44 mcg), both produced in the U.S., and the follow-on products Juntab^®^ (Mexico) and CinnoVex^®^ (Iran), both 30 mcg preparations, and the 44 mcg presentations Clausen^®^ (Uruguay) and Blastoferon^®^ (Argentina), these latter examples revealing considerable heterogeneity in immunochemical analyses and in “reporter gene assays” among the follow-on products but not in the brand medications. These studies also demonstrated significant pharmacological and biological potency differences between the innovators and the follow-on products [49]. Studies have consistently shown that lack of clinical data, confounded with absence of demonstration of bioequivalence and interchangeability of biosimilars, do not provide at present time evidence for their efficacy and safety. The economic impacts on individual and public health offered by the follow-on products have not been reflected in significant savings for the health systems [50]. Several international initiatives have developed, like the one promoted by the Latin American Committee for Treatment and Research in MS (LACTRIMS) [51], that encourage practitioners, MS study groups, and MS patient associations (most affiliated to the MSIF) from the region (20 countries) to coordinate with local health officials providing information and education on the licensing process, and even participating as independent advisors, striving for the proposed non-innovative follow-on medications to provide adequate clinical efficacy and safety data.

## 6. Conclusions

Except for rare exceptions, MS has in fact become a global disease affecting virtually every ethnic and racial group. The widespread epidemiologic presence of the disease has carried tremendous socioeconomic challenges due to the limitation of access and barriers to MS management, notably in countries still undergoing economic development. Considering that comorbidities (obesity, hyperlipidemia, migraine, rheumatological conditions) have been reported to increase the risk of relapse in MS [52], emphasis in management of these comorbidities, including a healthy diet and exercise, should be part of the management paradigm across the globe. Ensuring improved diagnosis, access to treatment, information, and available support resources require coordinated efforts from local and regional neurological MS study groups, societal MS organizations, and patient support groups. The 2017 McDonald panel recognizes this need and encourages MS diagnostic validation in non-Western European ethnicity populations (since 2000, the diverse revisions have applied practically to only Caucasian populations), and to geographic areas where the disease has a low prevalence. Revisions to the MS criteria are conducted every 5–7 years, once new or more advanced diagnostic technology and documented clinical data justify updating the diverse criteria of the proposal. It is expected the next revision will include contributions from the international committees for treatment and research in MS from all areas of the world. Tangible and indirect expenses compound the associated costs of necessary but complex multidisciplinary MS care. In this commentary, these aspects are reviewed from an international perspective while providing awareness and potential paths to alleviate these actual concerns, including addressing the concern of insufficient data on follow-on therapeutic molecules.
